# Maternal and newborn health needs for women with walking disabilities; “*the twists and turns*”: a case study in Kibuku District Uganda

**DOI:** 10.1186/s12939-019-0947-9

**Published:** 2019-03-12

**Authors:** Rebecca R. Apolot, Elizabeth Ekirapa, Linda Waldman, Rosemary Morgan, Christine Aanyu, Aloysius Mutebi, Evelyne B. Nyachwo, Gloria Seruwagi, Suzanne N. Kiwanuka

**Affiliations:** 1 Department of Health Policy Planning and Management, Makerere University School of Public Health, P.O.Box 7072, Kampala, Uganda; 2 Institute of Development Studies, Library Road, Brighton, BN1 9RE UK; 3 Department of International Health, Johns Hopkins Bloomberg School of Public Health, 615 N. Wolfe Street, Baltimore, MD 21205 USA

**Keywords:** Maternal, Newborn, Health needs, Women, Walking disabilities, Kibuku, Uganda

## Abstract

**Background:**

In Uganda 13% of persons have at least one form of disability. The United Nations’ Convention on the Rights of Persons with Disabilities guarantees persons with disabilities the same level of right to access quality and affordable healthcare as persons without disability. Understanding the needs of women with walking disabilities is key in formulating flexible, acceptable and responsive health systems to their needs and hence to improve their access to care. This study therefore explores the maternal and newborn health (MNH)-related needs of women with walking disabilities in Kibuku District Uganda.

**Methods:**

We carried out a qualitative study in September 2017 in three sub-counties of Kibuku district. Four In-depth Interviews (IDIs) among purposively selected women who had walking disabilities and who had given birth within two years from the study date were conducted. Trained research assistants used a pretested IDI guide translated into the local language to collect data. All IDIs were audio recorded and transcribed verbatim before analysis. The thematic areas explored during analysis included psychosocial, mobility, health facility and personal needs of women with walking disabilities. Data was analyzed manually using framework analysis.

**Results:**

We found that women with walking disabilities had psychosocial, mobility, special services and personal needs.

Psychosocial needs included; partners’, communities’, families’ and health workers’ acceptance. Mobility needs were associated with transport unsuitability, difficulty in finding transport and high cost of transport. Health facility needs included; infrastructure, and responsive health services needs while personal MNH needs were; personal protective wear, basic needs and birth preparedness items.

**Conclusions:**

Women with walking disabilities have needs addressable by their communities and the health system. Communities, and health workers need to be sensitized on these needs and policies to meet and implement health system-related needs of women with disability.

## Background

Globally, persons with disability constitute 15% of the world’s population, with 80% of the people with disabilities living in Low- and Middle-Income Countries (LMICs). The WHO notes that the prevalence of disability is increasing, rather than reducing, and that it disproportionately affects poor and marginalized communities because of aging populations, global increases in non-communicable diseases. [1]. It is thus noteworthy that the Sustainable Development Goals explicitly mention disability as part of Goal 10, which aims to reduce inequality and promote the inclusion of all. In Uganda, 13% of persons have at least one form of disability [2]. Disability may be defined as the consequence of an impairment that may be physical, cognitive, mental, sensory, emotional, developmental, or a combination of these [3]. Disability may also be regarded as a dynamic interaction between a person with a chronic health condition, activities of daily living, his or her ability to participate in important social roles, and the physical as well as social environment within which s/he lives [4].

The United Nations’ Convention on the Rights of Persons with Disabilities, ratified by Uganda, guarantees persons with disabilities the same level of right to access quality and affordable healthcare, including sexual and reproductive healthcare services [5]. However, even in countries where laws exist to reduce inequalities in access to services, most reports have shown that compliance is still low [6–9]. This access includes physical access to places where services are offered, such as in health facilities. In most LMICs, maternal health care services have been structured to meet the needs of able-bodied women, neglecting the special maternity care needs of women with walking disabilities (WWD) [7, 8]. This inadvertently multiplies the barriers experienced by this marginalized category of women and makes them more susceptible to maternal- and newborn-related morbidity [9].

In Sub-Saharan Africa, maternal mortality currently stands at 546 maternal deaths per 100,000 live births [10, 11]. Consequently many Sub-Saharan Africa countries have a steep road to scale towards achieving target 3.1 of the Sustainable Development Goals (SDGs), which aims to improve the maternal mortality ratio to less than 70 maternal deaths per 100,000 live births by 2030 [12]. Uganda has made great strides in reducing its maternal mortality rate from 438 deaths per 100,000 live births registered in the 2011 Uganda Demographic and Health Survey (UDHS) report to the current 336 deaths per 100,000 live births [13]. Corresponding with this progress, is the marked improvement in pregnant women attending four or more antenatal care visits. This increased from 48% in 2011 to 60% in 2016, while the number of births in health facilities also increased from 57% in 2011 to 73% in 2016 [13] and similar results from a study in Northern Uganda [14]. Therefore, in order to achieve a further reduction in maternal mortality rates, there is need to scale up utilization of maternal health care services especially among marginalized populations [15].

Most efforts to improve maternal health outcomes in Uganda have focused on improving access and quality of care for women, without regard for the special needs of marginalized populations, such as teenage mothers, WWD or the extreme poor [14]. Advocacy groups have worked with marginalized populations to enhance their ability to demand better maternal health services, and with policymakers to advance the Government of Uganda’s commitment to safe motherhood. Most of these efforts have, however, focused on teenage pregnancy for which Uganda has adequate data and stakeholder support.

Despite Uganda’s Constitutional acknowledgment of the rights of persons with disabilities and substantial policy provision, persons with disability still struggle to access health services [16]. Disability in any of its manifestations restricts the ability of individuals to participate in what is considered “normal” in their everyday societies [7]. For instance, according to Ganle, WWD want to receive institutional maternal healthcare, but they often find it difficult to travel to access skilled care. Other health service barriers they encounter include inappropriate physical health infrastructure and healthcare providers’ insensitivity, lack of knowledge about their maternity care needs, and negative attitudes towards them [9]. Frequently the needs of disabled women are poorly understood. Studies report that able-bodied persons hold the perception that WWD should be asexual and hence childless; and when they do have children they are often judged to be unable to care for their offspring [9].

Moreover, in Uganda, persons with disability are often not valued as marriage partners because of the extra social and economic burden they pose and the perception that they cannot contribute economically to household incomes [17]. However, studies have reported that persons with disabilities do strongly defend and exercise their reproductive rights despite their social and environmental constraints; currently the fertility rate of women with disabilities in Uganda is 6.3% as compared to the general population which is at 5.4% [13]. Many disabled women perceive having children as an investment for the future in terms of social support and labor provided by grown children [18]. There is therefore a clear need for maternal health services for WWD in Uganda.

Understanding the needs and the experiences of WWD is key in formulating interventions that support them. Assessing systems and socio-cultural support structures and barriers is also critical to designing flexible, acceptable and responsive health systems which are attentive to the needs of WWD and which will improve their access to care [17]. However, there is a dearth of research on the needs and experiences of Ugandan WWD as they access maternal and newborn services. This study therefore explores the maternal and newborn health-related needs of WWD in communities and health facilities in the Kibuku District of Uganda.

## Methods

### Design and study area

We carried out a cross sectional qualitative study in September 2017 using In-depth Interviews (IDIs) to explore the maternal and newborn health experiences of WWD who had given birth between September 2015 and September 2017. WWD were selected as the study participations because studies have shown that they are often marginalized and do not easily access maternal and newborn health services [9], [17].

This study was carried out in Kibuku District, which is located in the Eastern region of Uganda with a population of 202,033 people and with 52% of the population being female. People with walking disabilities aged over 2 years account for 4% of Kibuku’s population or 7, 109 people [19]. Kibuku’s predominately rural population is mostly engaged in crop farming, animal husbandry, petty trading, and brick making. Kibuku has two counties, namely Kabweri and Kibuku, 17 sub-counties and 86 parishes. This study was carried out in 3 sub-counties (Kibuku town council, Kibuku rural, and Goligoli) as it was part of a larger study to inform a community scorecard intervention.

### Study methods, selection of study participants and data collection

We conducted 4 in-depth interviews (IDIs) with purposively selected participants in the sub-counties of Kadama, Kibuku rural, and Goligoli. Through the District Health Office, the Village Health Team (VHT) coordinators of the three different sub-counties were asked to identify any WWD who had given birth between September 2015 and September 2017. Only four WWD who fitted our desired criteria were identified from all the three sub counties. The reason we restricted selection to WWD who had given birth two years before the study date was to take advantage of the relatively fresh MNH experiences and to reduce potential retention bias among the participants. The IDIs were conducted within the participants’ homes to explore the MNH needs of WWD. We recruited two experienced female research assistants who had a good working knowledge of English and of Lugwere (the local language) and trained them in data collection. Investigators also actively participated in the data collection process. We translated the IDI guide from English to Lugwere, then back-translated and compared to ensure consistency and pre-tested the IDI guide in Kampala district, Central Uganda. We received written consent from participants to participate in the study and to audio record interviews that lasted on average two hours.

### Data management, analysis and ethical consideration

The research assistants transcribed the IDIs verbatim taking care not to alter meaning since the interviews were in the local language. We analyzed the data manually using framework analysis [20, 21]. The typed transcripts were each read several times and a thematic framework was developed based on psychosocial, mobility, health facility and personal needs. We then systematically applied this framework to each of our transcripts and sifted, charted and sorted material according to key issues and themes. Quotes from the transcripts that elaborately illustrated meanings or key message from the analysis were also identified.

Ethical clearance was obtained from the Makerere University School of Public Health Research and Ethics Committee (MakSPH HDREC) and approval from the Uganda National Council of Science and Technology (UNCST), study number SS 4323. Permission to carry out the research was further sought from the Kibuku District Health Office. The objectives, benefits and risks of the study were explained to the study participants and written informed consent obtained from all the four WWD. All data obtained during the study were treated as confidential and anonymous identifiers were used. We restricted data access to only the investigators and the two research assistants.

## Results

Our results are presented in the four main thematic areas derived through inductive thematic analysis of the interview transcripts exploring MNH needs. These include:-psychosocial, mobility, health facility and personal needs.

### Interview participants

Four rural mothers with walking disabilities were interviewed. Three of the four women were unmarried and unemployed, while one had an informal business. All the women had little or no education and, while their average age was 33.8 years, they ranged from 27 to 45 years old. The Table 1 below summarizes the characteristics of each interviewee.

**Table 1:** Background characteristics of the interviewees

	Participant I	Participant II	Participant III	Participant IV
Age	28	45	27	35
Marital status	Unmarried	Married	Unmarried	Unmarried
Level of education	No education	No education	Primary two	Primary two
Residence	Rural	Rural	Rural	Rural
Distance to nearest health facility	6 Kilometers	5 Kilometers	10 Kilometers	4 Kilometers
Occupation	None	None	None	Informal business
Disability	One arm and leg impaired	Both legs impaired	Both legs impaired	One leg impaired

### Psychosocial MNH needs of women with walking disabilities

Women with walking disabilities expressed a number of psychosocial needs during pregnancy, delivery and the postnatal period. These included acceptance by: partners, families, communities and health workers.

### Partners’ acceptance

The three unmarried WWD reported that their partners did not accept any responsibility during pregnancy and delivery. As is evident in the quotes below, they explained that the men who impregnate WWD did not wish to be identified as the fathers of the children. Partners sometimes recommended abortion in order to avoid paternal responsibility. As these mothers explained to us:

*There is nothing wrong with producing [children] but it would be good if the people who impregnate me take care of the pregnancy until delivery. But you find that they disappear and don't provide for you when you are pregnant and, even after delivery, no-one buys [you] even a piece of soap (WWD).*

*I have five children, but from three different men. The first man told me that there is nowhere he can take a disabled woman. The father of the second child got another woman who said ‘that disabled woman should not come to my home’. Then the father of this child said he will be supporting me, but I should stay here at my parents’ home (WWD).*

In contrast to the experiences of WWD who are unmarried, the married WWD reported acceptance and support from her husband during pregnancy, delivery and after delivery.

*When it was delivery time, we used to go together; he [my husband] could escort me because usually when you reach the facility there are certain things the providers may need during delivery so you need to have someone around you who can run up and down and to bring those items* (WWD**)**

### Families’ acceptance

The WWDs highlighted the need for acceptance from their natal families in order to help with their psychosocial needs, but also to provide material support during pregnancy, delivery and post-delivery. Some of these women also asked the babies’ paternal relatives to provide support, even though the fathers had denied responsibility for the pregnancies. The married woman found that she had a lot more family acceptance and support compared to the three unmarried women.

### Communities’ acceptance

The communities viewed these WWD as people who had no rights to sex or to bear children. This was emphasized in expressions of stigma, which – as shown in the quotes below – isolated WWD by refusing to give them casual work opportunities and shamed those men who consorted with them. As a consequence, these men were ashamed of their actions and, as shown above, denied involvement.

*I cannot earn because when I go to ask for some casual work people fear to give me saying ‘I cannot give a disabled person work in my garden because she will work while cursing [speaking bad prophesy about someone] (WWD).*

*He [my partner] said: how do people look at me moving with a disabled woman when we are going to the facility? … If they [health service providers] want they can attend to you, if they don't want [to attend to you as a single woman] let them leave but I cannot go there with you (WWD).*

### Health workers’ acceptance

The health workers were, in some cases, receptive and spoke positively to WWD; however, in other cases health workers were reported to make stigmatizing comments. According to the participants, health workers made it clear that, as far as they were concerned, WWD should ideally not have sex, and definitely not get pregnant. As is evident from the quotes below, the health workers’ reasons for this judgment included misguided empathy, but also their conviction that these women were an extra burden to their natal families since they were disabled and therefore unable to take care of their babies’ needs.

*During my first pregnancy the health workers used to ask me many questions like; ‘were you just raped or what?’ They thought maybe drunkards had raped me … Perhaps because I am disabled, they would not expect me to get pregnant (WWD).*

*The first time when I went to the facility, they told me that they wanted [to see] my partner. So I came back home without being attended to (WWD).*

*Perhaps because am disabled, they would not expect me to get pregnant. I think that is what came into their minds. They could not tell me the reasons why [they asked so many the questions], but I think it is my disability that made them think like that … I did not feel well because inside me I said ‘so these people [the health providers] don't expect me to get a normal or sound man that can marry me and they don’t expect that we can produce children because I am disabled?’ I would not tell them openly but it would remain in me (WWD).*

The above evidence shows the need to address the psychosocial needs of WWD, including partners, families’, communities’ and health workers’ acceptance of their disability, sexuality, desire for and experience of motherhood.

### Mobility-related needs of women with walking disabilities

The main mobility-related needs were associated with transport, child-care and health system-tasks, as presented in detail below.

### Transport-related needs

The mothers with walking disabilities reported three major concerns related to transport, which were the suitability of transport, the difficulty in finding transport and the high costs involved.

#### Suitability of transport

While most women in the villages walked to health facilities, WWD could not walk long distances and, as a result, had to use the commonest means of transport, namely motorcycles. As motorcycles are high off the ground, it was difficult for WWD – who were either heavily pregnant or who had recently given birth – to get on and off. In addition, WWD were unable to support themselves and carry their babies on the motorcycles at the same time. They therefore required help with getting on and off the motorcycles. As a result, each woman had to have somebody to accompany her and carry the baby once the women had given birth, oftentimes using a second motorcycle. The costs of hiring two motorcycles and the challenges of finding people to accompany them, especially in the light of fathers’ refusals to do this, made this a major hurdle to overcome. WWD opted instead to get to the clinics on their own initiatives and, as the following quote illustrates, this could involve massive personal struggle:

*I used to crawl up to Kibuku Health Centre...on the Maram road. I would leave here in the morning and reach the facility at around 1:00 pm because the distance is quite long [10km]*. (WWD)

#### Difficulty finding transport

The unpredictable timing of the need for transport to a health facility exacerbated the problem of accessing transport, especially for WWD residing in rural areas. If labor pains started at night, for example, it made it difficult for the mother to find transport as echoed below.

*I started getting labor pains at night and I had no one to take me to the facility so I delivered alone from here [at home]. After pushing [and delivering] the baby, I sent one of the children I was sharing the house with to my brothers … that is when my sister in-law came and helped me cut the cord. (WWD)*

#### High costs of transport

The high costs of transport were also challenging for WWD. This was not only linked to their need to hire two vehicles. Women also reported that motorcycle drivers’ charged more for the physically disabled as they viewed them as requiring more of their time and support.

#### Mobility problems limit the disabled from engaging in business

Participants stated that they were unable to engage in informal businesses in the villages – which is how other women make money – as it necessitated travelling long distances to shop and carry goods back home to sell. It was therefore difficult for WWD in the study to make money that could help them during pregnancy, delivery and postnatal period. Unmarried WWD, or women whose partners rejected them during pregnancy, had increased economic needs as they had no financial support from their partners.

### Health facility MNH needs of women with walking disabilities

The mothers with walking disabilities expressed some infrastructural and special service needs at the health facilities. These infrastructural needs included: lower examination and delivery beds, seats, ramps, and sanitary facilities, while special MNH services included: outreach services for MNH, shorter waiting times at the facility, and responsive health services. We provide further discussion of these findings below.

### Infrastructural needs

#### Examination and delivery beds

The mothers with walking disabilities reported that health facilities had no beds adapted to their conditions. The beds used for examination, delivery and maternity rest were too high for them and they reported difficulty getting on and off these beds. If they were to use the beds, they needed somebody to help, which was not always available especially in the case of unmarried WWD who also experienced far less or no partner support.

*The health provider told me to enter the examination room but when I reached it … the bed was high, I could not manage to climb it so I remained seated on the floor and I was examined on the floor. (WWD)*

#### Seats

Participants also reported that the seats in the service waiting areas were too high for them. If they wanted to sit down, they would have to find someone to help lift them onto the seats. As a consequence, they sat down on the floor while women without walking disabilities sat on chairs as they waited to be seen by a provider.

*The waiting area had high cemented seats … when I had a person to help me climb it I would climb and sit up like other mothers, but when I had no-one to help me to climb, I would sit down on the floor and wait for my name to be read, then crawl to the examination room. (WWD)*

#### Access routes

Respondents reported experiencing difficulty in using stairs to access buildings in the health facilities. The absence of access routes, such as ramps, made it difficult for WWD that used a wheelchair or crawled to enter the health facility buildings:

*There were difficult places to climb like at the entrance where there are steps, by then they had not put this inclined walkway [ramp] where you can just move without difficulty. So I would reach where the steps were and lift one knee and put it on one step and do the same to the other. I would struggle until I entered inside* (WWD).

#### Sanitary facilities

Respondents described the sanitary facilities in the health facilities as being completely inappropriate for their conditions and inaccessible. For example, there were no latrines designated or adapted for the WWD. The latrines and bathrooms they used were shared with women without walking disabilities and were often very dirty. This was particularly challenging for women who could not walk upright:

*You would find the latrines dirty, but I had nothing to do [implying she had no choice but to use the latrine in that dirty state] I would crawl in that messed-up place like that. The facility does not have separate latrines for disabled people... (WWD)*

### Responsive health facility MNH services

Respondents also expressed a need for special outreach services for antenatal and postnatal care. One of the WWD mentioned, with grateful appreciation, a particular health worker from one facility who used to visit her in her home and immunize her children whenever she had a newborn. The others, however, had to transport their children to the clinic, experiencing all the above-mentioned challenges in so doing.

#### Shorter waiting time at facilities

Respondents also expressed the need to be offered services as soon as they arrived at health facilities instead of queuing with the rest of the mothers. Shorter waiting times were needed because they sometimes felt pain when they sat for too long, and because travelling to and from the health facility took much longer, especially if they had to limp or crawl home.

*Also the waiting time was long because I would reach the facility at around 1:00 pm but come back here at 6:00 pm. Health workers would not consider my disability and attend to me first. I would follow the queue until they reach my book and call me to the examination room. (WWD)*

### Personal MNH needs of women with walking disabilities

Research into MNH care seldom focuses on needs that lie beyond the ability of the health system to provide. However, for WWD, these needs are accentuated by their disabilities and by the ways in which they interact with the health sector. We present these finding following categories of personal protective wear, basic needs and birth preparedness items and childcare-related needs.

#### Personal protective wear

Mothers with walking disabilities required personal protective wear for their hands and feet during movement to the facility and also when accessing the often-dirty sanitary facilities in the health facility. This need for protection for knees and hands was most urgent for those women who had both legs disabled and could only move by crawling.

*When going to ease myself I need to have protective gears like gloves and sandals to put on because I use my hands to move to these dirty places. (WWD)*

#### Basic needs like clothing, food and hygiene materials like soap

Because of their mobility related constraints, these mothers with walking disabilities found it hard to secure income-generating work and therefore were unable to afford basic supplies. While they were able to make do much of the time, when pregnant their basic needs were amplified and they required support with food, clothes and other hygiene materials to help them during pregnancy, delivery and post-natal care. This included baby clothes, baby sheets, razors, gloves, dresses, a basin and washing soap.

#### Childcare-related needs

In cases where women with walking disabilities had a supportive families or husbands, it was possible for them to find additional help and support. In such instances, childcare-related needs, including postnatal care, could be easily met. However, where the WWD were unmarried and had no supportive family members, they had difficulty in meeting these needs.

*If my child falls sick, it is my mother who has to struggle to look for money to treat them. Not only that, but she has to carry the child to the facility for treatment because I cannot walk while carrying a baby. (WWD)*

*You need to get support when you want to carry the baby because, like me now, when I am a mother who has newly delivered, I cannot support my baby when breast feeding, even bathing the baby I cannot. There has to be a person to help me put the baby on my lap and also support [the baby] as I breastfeed because at that stage the baby is so delicate that I cannot lift him or her with only one hand (WWD).*

## Discussions

Our study findings have emphasized that WWD have specific MNH-related needs that want attention by the health system. These include psychosocial, mobility, health facility-related, and personal needs. In this discussion, we focus on these needs and their implications for these WWD.

### Psychosocial MNH needs of women with walking disabilities

Women with walking disabilities require acceptance by close relatives, such as their natal families and partners, as well as acceptance by their respective communities and health service providers. This acceptance shapes the manner in which families, partners and health workers respond to the MNH needs of WWD. Our work showed that WWD are often discriminated by their natal families, partners and health workers. This has been attributed, not to the disability itself, but rather to the communities’ inabilities to understand the disabilities as well as to religious and cultural negative beliefs about disability [22, 23]. The findings in our study were similar to findings from Zambia and United States, where some members of the community tended to think that WWD should be asexual, not get pregnant and not get married [24, 25]. Because of the stigma attached to WWD getting pregnant, men tended to shun WWD [25, 26], while those who had relationships that resulted in pregnancies tended to deny their responsibilities [27]. This lack of acceptance therefore restricts WWD from fulfilling their sexual desires, from bearing children, and from bringing them up with paternal influence; yet there is no reason to expect that women with disabilities’ desire to have children is any different from that of women without disabilities [24, 25].

When these women get pregnant, they need support right from the start of pregnancy until, and after, delivery time [28, 29], as do able-bodied women. They often require even more emotional support than women without disabilities given the stigma that surrounds them [30]. However, the lack of family and partner acceptance often limits support with basic needs and mobility during pregnancy, making it difficult for WWD to cope during pregnancy. In addition, they may need an extra person to carry their child after delivery because they cannot sit on the motorcycles while carrying their babies. In the early days of life, the babies are delicate and the women’s lack of mobility also makes breastfeeding and bathing the babies very difficult. A study carried out in Nigeria among women without walking disabilities found that support from spouses and significant others was relevant in boosting exclusive breast feeding [31]. Clearly WWD need additional support to encourage exclusive breastfeeding. Moreover, WWD should be supported to participate in programs that can improve their financial preparedness. Women support groups have yielded some positive results in Jharkhand and Orissa [32].

Not only do women with disabilities face challenges of acceptance in communities, they also face this challenge in health facilities. Existing evidence has shown that health workers are sometimes insensitive to the needs of WWD [33]. [9], [34, 35] and [18]. Studies in Kenya and Uganda showed that WWD felt disrespected by service providers [30]**.** Such an attitude could cause women with disabilities to shun institutional MNH services.

### Mobility-related needs

Women with disabilities face several challenges related to mobility. They are often unable to travel to the market, health facility or even possible workplaces. This limits their access to health facilities and access to employment [37–39]. Indeed studies have shown that many WWD are unemployed [40–44]. Access to health facilities is further constrained by factors such as long distances [38], [18] and the high cost of transport [39]. Many disabled women are either unemployed or lack family support hence affording transport money is often a challenge [40]. The poor suitability of transport, such as motorcycles, further compounds the transport problem. Most rural communities in LMICs use motorcycles, bicycles or walk on foot [41]. However, the motorcycles and bicycles are high and getting onto, or off, them is difficult for WWD [42]. In such situations, those who have support from their families or partners may be more able to use motorcycles than those who do not. Motorcycle riders are often unwilling to bear the extra burden, and possible stigma, of carrying such clients.

In more developed countries as opposed to LMICs, many people with walking disabilities can use public transport because transportation is more accessible for them [36, 43]. This has been made easier with the aid of new assistive technology such as robots to help people with disabilities get to couches, or vehicles which can be lowered [44]. In less developed countries where these initiatives are absent [18], taking services closer to WWD, for example through integrated community-based interventions, can benefit both people with and without walking disabilities, and optimize resource use [45]. Local innovators in developing countries should also explore the development of low-cost assistive devices that can ease the mobility needs of WWD.

### Health facility MNH needs of women with walking disabilities

Studies carried out in Nepal and Uganda showed that health workers not only have a negative attitude towards WWD, they are also not trained to handle WWD [17], [8]. Women with disabilities are sometimes unable to fulfill facility requirements and therefore need to be given special consideration. For example, while many facilities in Uganda require women to come to the facilities with their partners, coming with partners to the health facility for Antenatal Care (ANC) is not easy for WWD because of the poor acceptance by their partners [27]. Our work shows that, in some instances, these WWD are sent back home without a service for this reason. Such responses only exacerbate their difficulty in accessing MNH services and health care workers should be more accommodative in such circumstances. Moreover, to encourage the involvement of male partners, health facilities could consider issuing a special invitation to the partners of WWD. Some African studies showed that inviting partners using a written document improved their ANC attendance [46–49].

Another area where special consideration by health workers is called for is related to households’ ability to prepare adequately for birth. As noted above, rural WWD face many challenges with regard to finding employment [47], which in turn would allow them to earn money to purchase the necessary requirements for the newborn and mother at delivery [48]. When WWD lack birth-preparedness items like clothes for the baby, gloves, etc., the health care workers need to take their economic situation into consideration. In view of these multiple challenges faced by WWD, health providers should provide special consideration, for example, they could be prioritized at health facilities and seen first instead of being expected to wait in a long queue. However, according to Groce et al*,* health professionals do not often plan for the special needs of people with disabilities [49]. Work done in Uganda showed that long waiting times discouraged people with physical disabilities from accessing health facility services [17], [18].

### Health facility infrastructure needs

Our study revealed that the infrastructure in many facilities is inappropriate for WWD. For example, many facilities in Kibuku do not have ramps for people with disabilities. Similar findings have been reported in a study done in Kampala, where the absence of ramps and wheelchairs made services inaccessible for people with disabilities [17]. Access is even worse where there are stairs. Delivery and examination beds are often too high for the women to climb onto; health care workers therefore end up performing procedures like examinations and deliveries on floors, which are not aseptic. This predisposes women with disabilities to infection. Findings from studies in Kampala and Bangladesh found similar results [17] [49]. Such actions may reduce client satisfaction with services and could result in decreased use of formal services and more use of alternative providers such as traditional birth attendants or TBAs [50]. Similarly, the seats in health facilities are often so high that the WWD are not able to use them without assistance and yet there are no wheel chairs for them [17]. They therefore resort to sitting on dirty floors while other women sit on chairs or benches. This reinforces the stigmatization of WWD while also making them feel inferior to their colleagues and potentially further discouraging them from using health facilities.

The same challenge is faced with sanitary facilities. In our study WWD who do not have the use of their lower limbs reported that they have no special facilities and often have to crawl into very dirty toilet facilities used by the other patients. This increases the risk of infection and deters them from seeking services in formal health facilities. These findings correspond to studies done elsewhere, which have also revealed that people with disabilities experience more hardship with regard to accessing sanitary facilities [51], [17] [52]. Health providers should make a special effort to provide relevant infrastructure such as ramps, seats, beds and latrines, which are designed to enable WWD to utilize health service-related infrastructure. In countries where separate facilities are not available, protective wear could be provided to enable them have some protection while they access the latrines or to ensure that medical procedures take place in aseptic conditions.

### Limitations of the study

The study had a small sample size because we only found four WWD who had given birth in the last two years from the date of the study in the study area. The study was a single method study, which used only IDIs. However this was deemed an appropriate fit for the study and suited the respondents since the study focused on the MNH experiences of WWD.

## Conclusion

Women with disabilities have several needs that include psychosocial and personal needs; mobility-related needs, as well as health facility-related needs. More sensitization to counter the stigmatization and to educate citizens about the special needs of WWD should be undertaken, followed by advocacy that can encourage decision-makers to explore, innovate and put in place mechanisms to meet their special needs. This sensitization should target families and communities, as well as health care providers and policy makers. This will enable their partners, families and communities to understand the problems that they face and the kinds of support that they require. Health care providers and policy makers should also dedicate more time and resources to providing training to health workers so that they can provide services that are responsive to the needs of WWD. Lack of support by health workers can drive such women to deliver with TBAs, whereas health care providers’ support can enhance uptake of formal health care services.

Policy makers and health care providers should take deliberate actions to provide appropriate infrastructure. Initially, they could select a few facilities that can be made disability-friendly by providing the required infrastructure such as ramps, assistive devices for climbing high beds and seats. They can also explore expanding the target group for outreach services to include WWD, so that they can receive health services closer to their homes and thereby reduce arduous travel. Lastly, we suggest that policy makers broaden partnerships with NGO’s who can provide opportunities for equipping people with disabilities with skills that can enable them find employment or start their own small business that can enable them to meet their personal needs.

## Abbreviations

ANC: Antenatal care
IDIs: In-depth interviews
LMICs: Low and middle income Countries
MakSPH HDREC: Makerere university school of public health higher degrees research and ethics committee
MNH: Maternal and newborn health
NGOs: Non -government organizations
SDGs: Sustainable development goals
TBAs: Traditional birth attendants
UDHS: Uganda demographic and health surveys
UNCST: Uganda national council of science and technology
VHTs: Village health teams
WWD: Women with walking disabilities
